# Dopamine and Methamphetamine Differentially Affect Electron Transport Chain Complexes and Parkin in Rat Striatum: New Insight into Methamphetamine Neurotoxicity

**DOI:** 10.3390/ijms23010363

**Published:** 2021-12-29

**Authors:** Viktoriia Bazylianska, Akhil Sharma, Heli Chauhan, Bernard Schneider, Anna Moszczynska

**Affiliations:** 1 Department of Pharmaceutical Sciences, Wayne State University, Detroit, MI 48201, USA; viktoriiabazylianska@wayne.edu (V.B.); akhilsharma@wayne.edu (A.S.); helichauhan@wayne.edu (H.C.); 2 Brain Mind Institute, École Polytechnique Fédérale de Lausanne, School of Life Sciences, CH-1015 Lausanne, Switzerland; bernard.schneider@epfl.ch

**Keywords:** methamphetamine, dopamine, electron transport chain complexes, parkin

## Abstract

Methamphetamine (METH) is a highly abused psychostimulant that is neurotoxic to dopaminergic (DAergic) nerve terminals in the striatum and increases the risk of developing Parkinson’s disease (PD). In vivo, METH-mediated DA release, followed by DA-mediated oxidative stress and mitochondrial dysfunction in pre- and postsynaptic neurons, mediates METH neurotoxicity. METH-triggered oxidative stress damages parkin, a neuroprotective protein involved in PD etiology via its involvement in the maintenance of mitochondria. It is not known whether METH itself contributes to mitochondrial dysfunction and whether parkin regulates complex I, an enzymatic complex downregulated in PD. To determine this, we separately assessed the effects of METH or DA alone on electron transport chain (ETC) complexes and the protein parkin in isolated striatal mitochondria. We show that METH decreases the levels of selected complex I, II, and III subunits (NDUFS3, SDHA, and UQCRC2, respectively), whereas DA decreases the levels only of the NDUFS3 subunit in our preparations. We also show that the selected subunits are not decreased in synaptosomal mitochondria under similar experimental conditions. Finally, we found that parkin overexpression does not influence the levels of the NDUFS3 subunit in rat striatum. The presented results indicate that METH itself is a factor promoting dysfunction of striatal mitochondria; therefore, it is a potential drug target against METH neurotoxicity. The observed decreases in ETC complex subunits suggest that DA and METH decrease activities of the ETC complexes via oxidative damage to their subunits and that synaptosomal mitochondria may be somewhat “resistant” to DA- and METH-induced disruption in mitochondrial ETC complexes than perikaryal mitochondria. The results also suggest that parkin does not regulate NDUFS3 turnover in rat striatum.

## 1. Introduction

Methamphetamine (METH) is a central nervous system (CNS) psychostimulant with a high potential for abuse. Consequently, METH abuse is a serious public health problem in the US and worldwide. In the US, over 1.2 million people currently use METH [1]. Alarmingly, there is no effective FDA-approved pharmacotherapy for METH addiction or preventive measures against toxicity of this drug to the brain [2]. Chronic METH use, particularly at high doses, can have severe physical and psychological consequences, including cognitive impairments and psychotic symptoms [3], which are the manifestations of this neurotoxicity. High doses of METH are particularly neurotoxic to the dorsal striatum where the drug induces dopaminergic (DAergic) deficits and increases the risk of developing Parkinson’s disease (PD) by as much as threefold compared with individuals who do not use the drug [4,5,6]. This enhanced vulnerability to PD may relate to the neurotoxic effects of METH to DAergic axonal terminals in the dorsal striatum that are similar to PD pathology [7].

**Figure 1:**
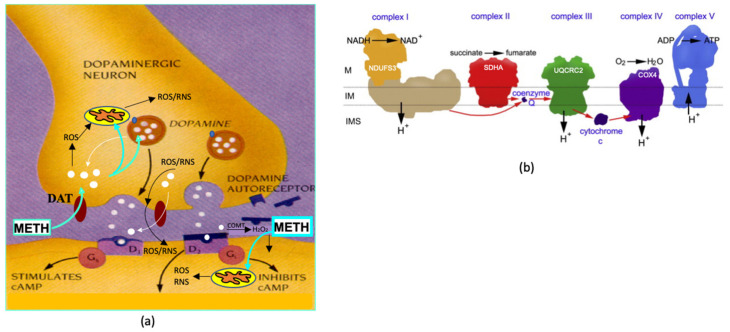
(**a**) Methamphetamine (METH) effects in the striatum. METH is taken up by dopamine (DA) transporter (DAT) and triggers the release of DA from the storage vesicles to the cytoplasm within DAergic terminals and subsequently to the synaptic cleft. Released DA quickly autoxidizes, producing several reactive oxygen species (ROS). ROS-damaged mitochondria produce more ROS and reactive oxygen species (RNS). Some DA-derived ROS and RNS diffuse to postsynaptic neurons and glia and affect mitochondria therein. (**b**) Electron transport chain (ETC) complexes (adapted from [28]) with localization of NDUFS3, SDHA, UQCR2, and COXIV subunits. Abbreviations: COMT, catechol-O-methyltransferase (catalyzes DA metabolism to hydrogen peroxide (H_2_O_2_)); IM, inner membrane; IMS, intramembrane space; M, matrix.

Production of cellular energy is enabled by the electron transport chain (ETC), located on the highly folded inner mitochondrial membrane. The ETC consists of five protein complexes (complexes I–V), each of which contributes to the generation of adenosine triphosphate (ATP) and release of energy (Figure 1A). Considering the high energy demands of neuronal cells, studying mitochondrial function in neurons occupies a central place in neurosciences. Beyond providing energy, mitochondria take part in a multiplicity of pathways that serve to regulate cellular life and death. Thus, dysfunction of mitochondria has been implicated in major neurodegenerative conditions, including PD [8] and METH neurotoxicity [7]. Despite substantial progress in the understanding of METH-induced damage of neuronal cells, a full understanding of the mechanism underpinning the toxic effect of the drug on the central nervous system has not yet been achieved. For example, the information on the effects of systemically administered METH on mitochondria localized in striatal axonal terminals is very limited [9,10,11,12]; therefore, more research on METH neurotoxicity to these mitochondria is warranted, particularly on METH neurotoxicity to complex I, as a deficit in activity of this mitochondrial enzymatic complex is one of the hallmarks of PD [8]. More research on METH neurotoxicity to perikaryal mitochondria is also needed, as existing literature reports are discrepant and appear to differ with the route of METH delivery, METH administration paradigm, and length of withdrawal from the drug.

Another understudied area in METH neurotoxicity to mitochondria is the neurotoxicity of the METH molecule itself. Being a positively charged molecule, METH can dissipate the electrochemical gradient within the mitochondria, induce oxidative stress, and decrease ATP synthesis [7,13]. Despite the potential of METH itself to be neurotoxic, there are no studies on the effects of METH alone on striatal mitochondria [14], and the mechanism by which METH molecule-induced oxidative stress contributes to cellular dysfunction is poorly understood. This is because it is difficult to separate METH effects from DA effects in vivo. The systemic administration of METH results in exposure of the striatum to both METH and DA. METH triggers a release of DA from the storage vesicles to the cytoplasm within DAergic terminals and subsequently to the synaptic cleft [15]. Released DA quickly autoxidizes, producing several reactive oxygen species (ROS). METH exposure also leads to production of reactive nitrogen species (RNS) [7]. Some DA-derived ROS and RNS diffuse to postsynaptic neurons and glial and affect mitochondria therein [16,17]. Figure 1B delineates METH actions in the striatum.

In vitro studies have demonstrated that parkin is a neuroprotective protein involved in maintenance of healthy mitochondria, including translation of several subunits and mitophagy [18,19,20]. Loss of parkin activity has been reported to cause deficits in respiratory complexes, particularly in complex I [21]. Both parkin dysfunction and complex I dysfunction have been implicated in the pathogenesis of PD [22,23]. Our in vivo studies have demonstrated that neurotoxic METH doses cause a decrease in parkin levels in striatal axonal terminals, while overexpression of parkin attenuates METH neurotoxicity in the striatum [24,25]. Based on the aforementioned data, we hypothesized that parkin has a role in maintaining complex I function in rat striatum.

The goal of the present study was three-fold: firstly, to tease out the effects of DA from the effects of METH on the levels of selected striatal mitochondrial subunits; secondly, to assess the susceptibility of synaptosomal (terminal axonal) vs. perikaryal mitochondria to oxidative stress induced by the DA and METH combination; and thirdly, to determine whether DA or METH decreases parkin levels and whether parkin has an effect on the levels of subunit NDUFS3 of complex I. We chose this subunit because it is crucial for complex I activity [26], and it is cleaved in response to depolarizing agents [12,27].

We provide in vitro evidence that METH alone can decrease the levels of a few striatal mitochondrial protein subunits, including NDUFS3 of complex I, and that the selected ETC subunits from axonal terminals do not decrease in response to DA and METH as do perikaryal mitochondria under our experimental conditions. We also provide in vivo evidence that administration of toxic METH doses does not result in a deficit in the levels of NDUFS3 1 h after the last dose of the drug and that overexpression of parkin does not regulate NDUFS3 levels in rat striatal synaptosomes.

Our study adds information to two major gaps in scientific knowledge in the field of METH neurotoxicity: the lack of information on the effects of METH alone on striatal mitochondria and the limited information on the effects of the DA and METH combination on mitochondria located in striatal axonal terminals. Overall, our data suggest that the METH molecule is a potential drug target in METH neurotoxicity and that parkin levels are not critical for activity of complex I.

## 2. Results

### 2.1. Effects of Dopamine on Cytoplasm-Suspended Striatal Mitochondria

**Figure A1:**
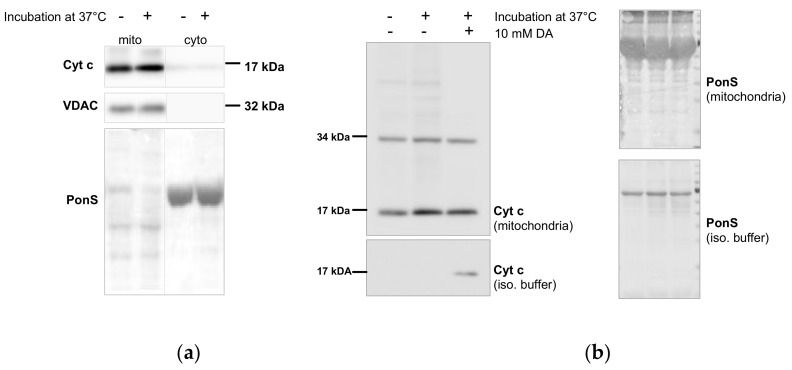
Effects of 30 min incubation at 37 °C on the integrity of (**a**) cytosol-suspended or (**b**) isolation-buffer-suspended mitochondria. Cytochrome c was not released either from the cytosol-suspended or isolation-buffer-suspended mitochondria after 30 min incubation at 37 °C. To confirm mitochondrial integrity during incubation, the levels of the VDAC were measured in the cytosol-suspended mitochondria; incubation did not change VDAC levels (**a**). In (**b**), a sample exposed to 10 mM DA served as positive control. Abbreviations: Cyt c, cytochrome c; DA, dopamine; iso. buffer, isolation buffer; PonS, Ponceau S (loading control); VDAC, voltage-dependent ion channel.

At millimolar concentrations, DA can depolarize mitochondria with the consequent release of cytochrome c [29,30]. In our mitochondrial preparations, DA-triggered release of cytochrome c was observed at >10 mM DA (Appendix A, Figure A1). To investigate how increasing concentrations of DA affect the levels of striatal mitochondrial ETC complexes, cytoplasm-suspended mitochondria were treated with 0–50 mM DA for 30 min at 37 °C. Following separation, mitochondrial and cytoplasmic fractions were examined for immunoreactivities of selected complex I, complex II, complex III, and complex IV subunits. We chose to investigate the NDUFS3 subunit of complex I, SDHA subunit of complex II, UQCRC2 of complex III, and subunit IV of complex IV, which we previously assessed in rats exposed to systemic binge METH [12]. The NDUFS3 subunit is important for the activity of complex I [26], while SDHA is important for complex II activity [31,32].

**Figure 2:**
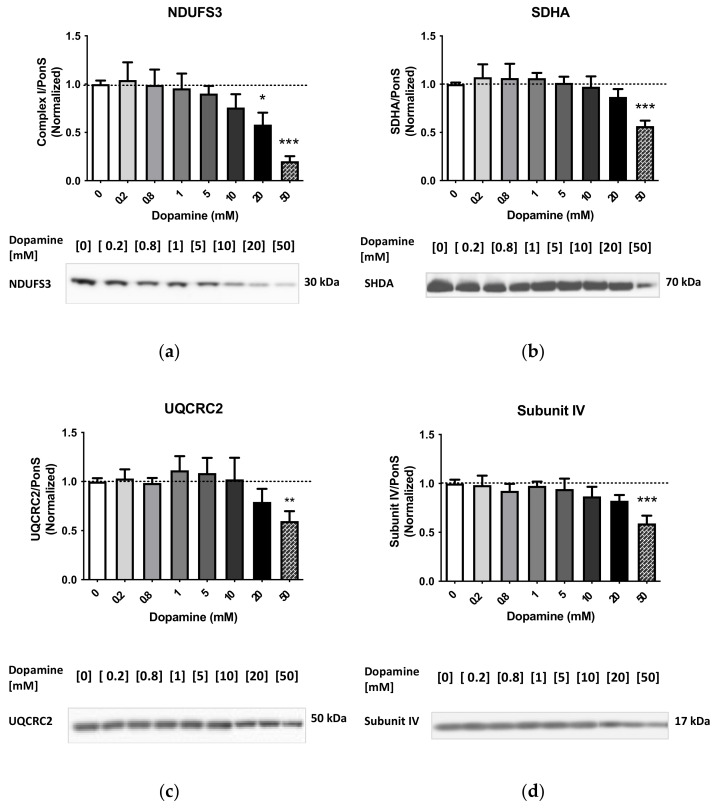
Effects of increasing doses of DA on the levels of subunit (**a**) NDUFS3 (complex I), (**b**) SDHA (complex II), (**c**) UQCRC2 (complex III), and (**d**) subunit IV (complex IV) in cytoplasm-suspended mitochondria. * *p* < 0.05, ** *p* < 0.01, *** *p* < 0.001, one-way ANOVA with Dunnett’s *post hoc* test.

**Figure A3:**
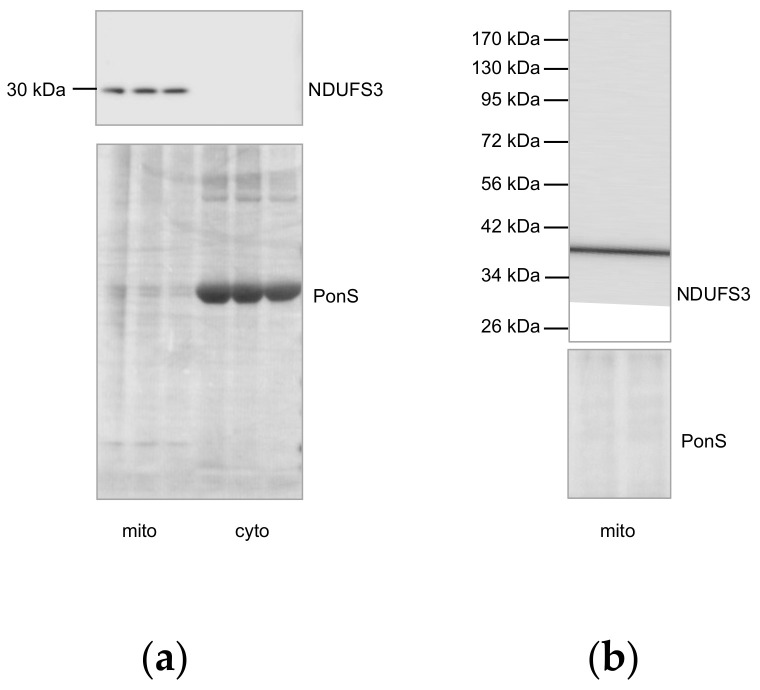
Cytoplasm-suspended mitochondria were incubated for 30 min with 10 mM dopamine (DA) at 37 °C. Mitochondrial and cytoplasmic fractions were subjected to SDS-PAGE under nonreducing conditions. Membranes were probed with anti-NDUFS3 antibody. (**a**) Lack of NDUFS3 immunoreactivity in cytoplasmic fraction. (**b**) Lack of NDFS3 immunoreactivity above 30 kDa. Abbreviations: cyto, cytoplasm; mito, mitochondria; PonS, Ponceau S (loading control).

The immunoreactivities of complexes I–IV were significantly decreased after incubation with 50 mM DA (NDUFS3: −80%, *p* < 0.001; SDHA: −44%, *p* < 0.001; UQCRC2: −40%, *p* < 0.01; subunit IV (COX4): −41%, *p* < 0.001; one-way ANOVA, followed by Dunnett’s multiple comparison *post hoc* test), suggesting disintegration of mitochondria at this concentration. All ETC enzyme immunoreactivities showed decreases at 20 mM DA, but only NDUFS3 immunoreactivity was significantly decreased after incubation with 20 mM DA (−42%, *p* < 0.05, one-way ANOVA with Dunnett’s *post hoc* test). At 10 mM DA, NDUFS3 immunoreactivity was decreased significantly when assessed by the Student’s unpaired two-tailed *t*-test (−24%, *p* < 0.05). The data are summarized in Figure 2 and Figure A3).

### 2.2. Effects of Methamphetamine on Cytoplasm-Suspended Striatal Mitochondria

**Figure 3:**
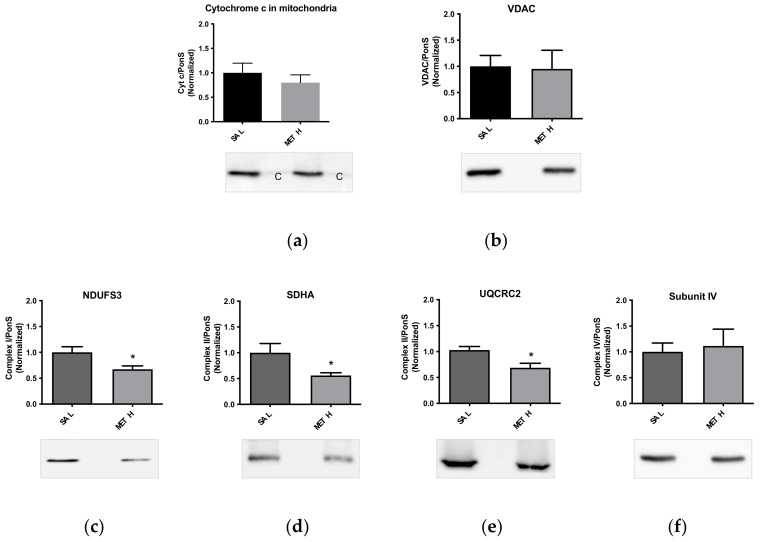
Effects of 10 µM METH on the levels of (**a**) cytochrome c, (**b**) VDAC, (**c**) NDUFS3 (complex I), (**d**) SDHA (complex II), (**e**) UQCRC2 (complex III), and (**f**) subunit IV (complex IV) in cytoplasm-suspended mitochondria. * *p* < 0.05, Student’s *t*-test. Abbreviations: c, cytoplasm; METH, methamphetamine; SAL, saline; VDAC, voltage-dependent anion channel.

**Figure A4:**
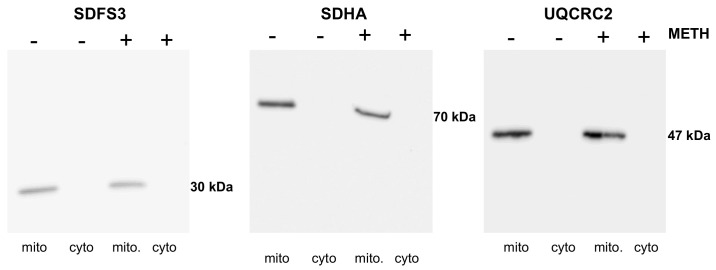
Cytoplasm-suspended mitochondria were incubated for 30 min with 10 µM methamphetamine (METH) at 37°. Mitochondrial and cytoplasmic fractions were subjected to reducing SDS-PAGE. Membranes were probed with anti-NDUFS3, anti-SDHA, or anti-UQCRC2 antibody. Lack of NDFS3, SDHA, or UQCRC2 immunoreactivity in cytoplasmic fraction.

To determine the effects of METH alone on the immunoreactivities of striatal mitochondrial complexes suspended in the cytoplasm, the preparations were incubated with 10 µM METH for 30 min at 37 °C. A trend toward statistical significance for a decrease in the immunoreactivity of cytochrome c was detected in METH-treated mitochondria (*p* = 0.07) (Figure 3a). METH did not decrease the immunoreactivity of the voltage-dependent anion channel (VDAC), a multifunctional housekeeping mitochondrial protein in cytoplasm-suspended mitochondria (Figure 3b). However, METH decreased the levels of the complex I subunit NDUFS3 (−23%), complex II subunit SDHA (−44%), and complex III subunit UQCRC2 (−31%) (*p* < 0.05, Student’s *t*-test) in these mitochondrial preparations (Figure 3c–e). The immunoreactivity of the complex IV subunit (subunit IV) was not significantly decreased by METH (Figure 3f). Please see Figure A4 for supplementary data.

### 2.3. Effects of Dopamine or Methamphetamine on Isolation-Buffer-Suspended Striatal Mitochondria

**Figure 4:**
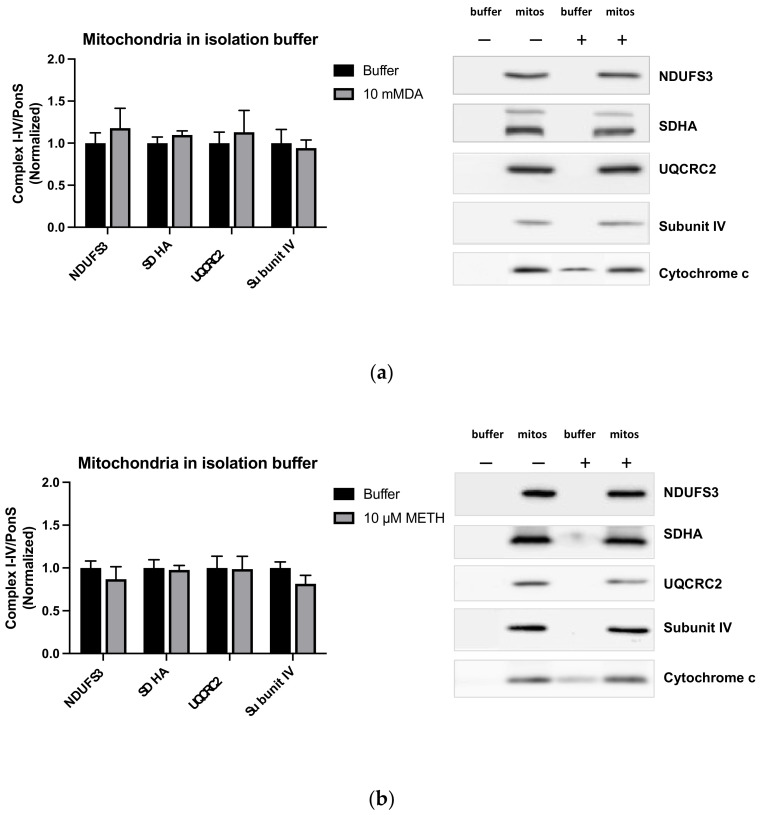
Effects of 10 mM DA (**a**) or 10 µM METH (**b**) on cytochrome c, NDUFS3 (complex I), SDHA (complex II), UQCRC2 (complex III), and subunit IV (complex IV) in isolation-buffer-suspended mitochondria. An increase in cytochrome c immunoreactivity was observed in the cytoplasm (Lane 3) after incubation of mitochondria with DA or METH. No significant changes were observed in the levels of mitochondrial subunits.

**Figure 7:**
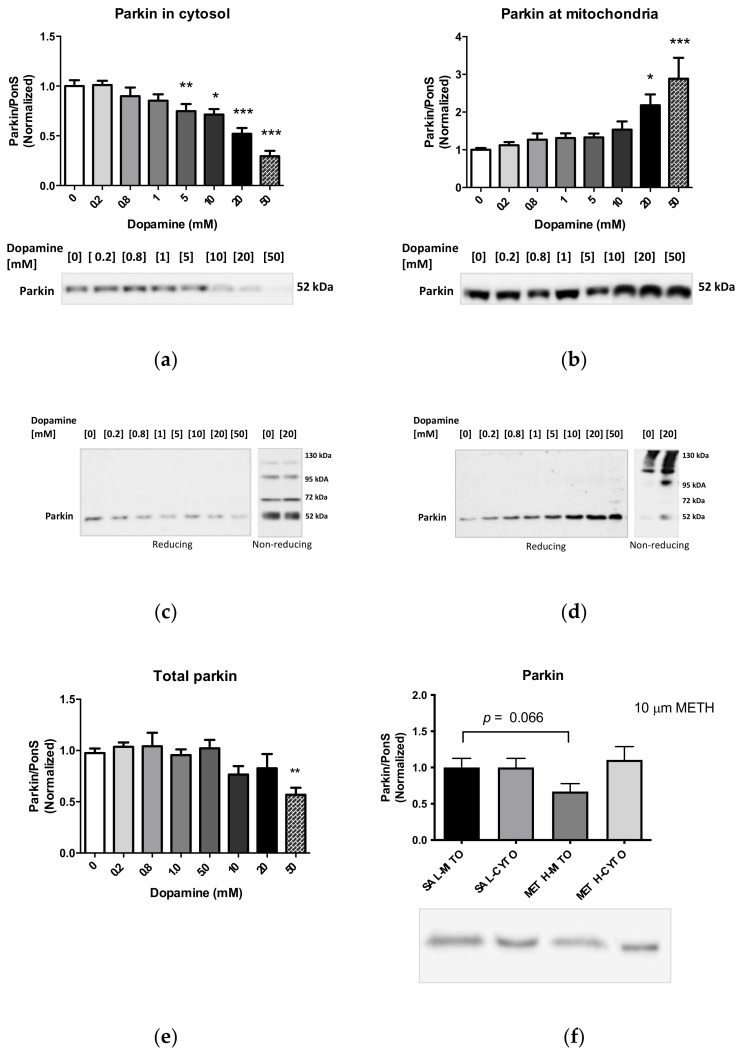

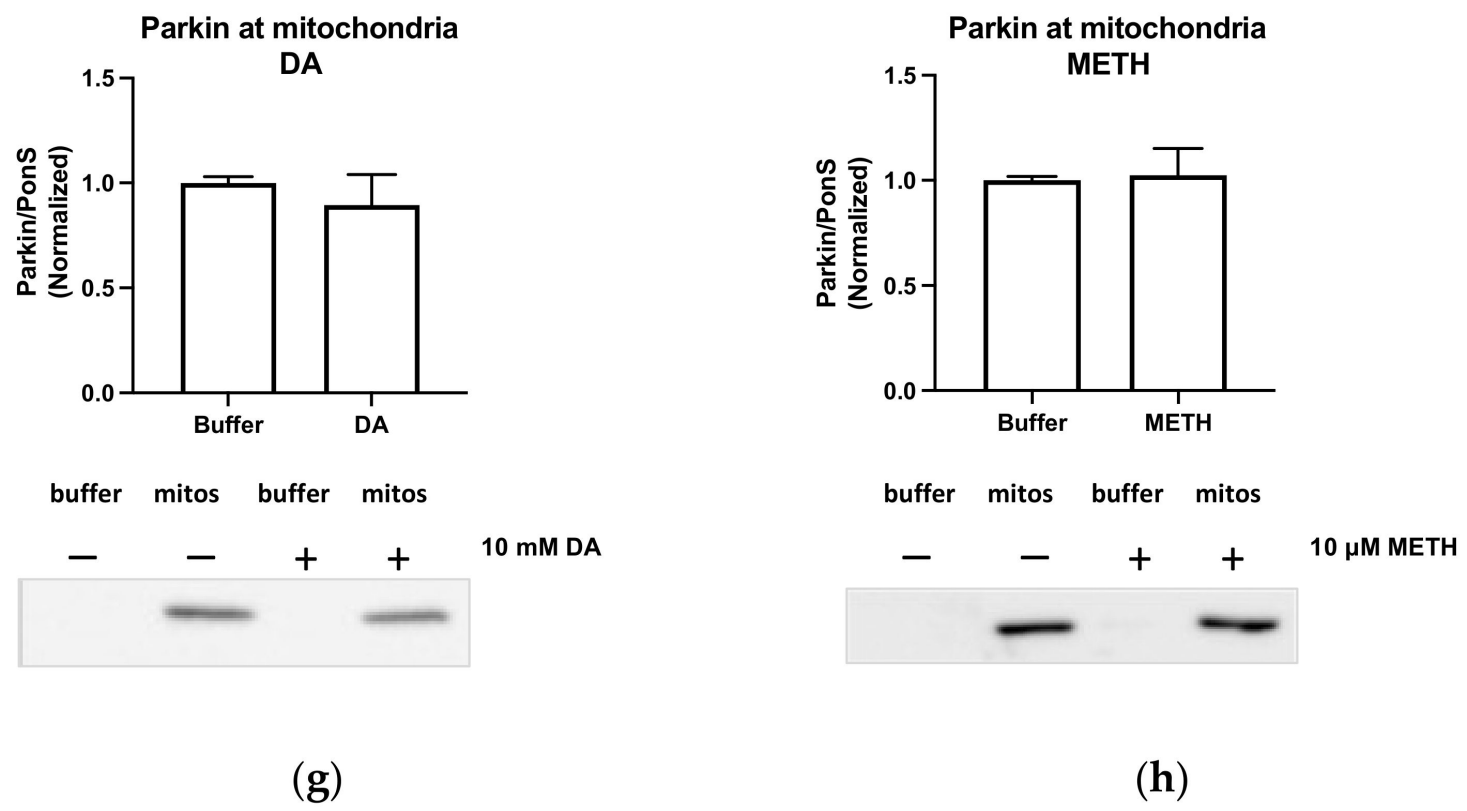
(**a**–**d**) Effects of increasing doses of DA on the levels of parkin in the (**a**,**c**) cytoplasmic and (**b**,**d**) mitochondrial fraction. High doses of DA significantly decreased parkin immunoreactivity in the cytoplasmic fraction while increasing it in the mitochondrial fraction under reducing conditions. Under nonreducing conditions (**c**,**d**), high DA doses caused aggregation of parkin in the mitochondrial fraction. (**e**) Total levels of parkin showed a trend toward statistical significance for a decrease. (**f**) The 10 µM METH decreased parkin immunoreactivity in the mitochondrial fraction but not in the cytoplasmic fraction. (**g**,**h**) The effects of 10 mM DA (**g**) or 10 µM METH (**h**) on the levels of parkin in isolation-buffer-suspended striatal mitochondria. * *p* < 0.05, ** *p* < 0.01, *** *p* < 0.001, one-way ANOVA with Dunnett’s *post hoc* test.

To determine whether cytoplasmic components change the effects of DA or METH on ETC enzyme immunoreactivities, intact striatal mitochondria were isolated from the striata, suspended in the isolation buffer, and incubated with saline, 10 mM DA, or 10 µM METH for 30 min at 37 °C. The DA concentration of 10 mM was chosen because the immunoreactivity of cytoplasmic parkin at this DA concentration was significantly decreased (see Figure 7 in the Section 2.6) to similar levels detected in vivo 1 h after binge METH (4 × 10 mg/kg) [24]. After DA or METH treatment, the immunoreactivity of cytochrome c in the isolation buffer increased (Figure 4). There was no change in immunoreactivities of the mitochondrial subunits after incubation with DA or METH (Figure 4), suggesting that the cytoplasmic components are needed for attenuation of NDUFS3, SDHA, and UQCRC2 by these molecules. The immunoreactivity of the VDAC is shown in Figure A3.

### 2.4. Effects of Dopamine and Methamphetamine Combination on Cytoplasm-Suspended and Isolation-Buffer-Suspended Striatal Mitochondria

**Figure 5:**
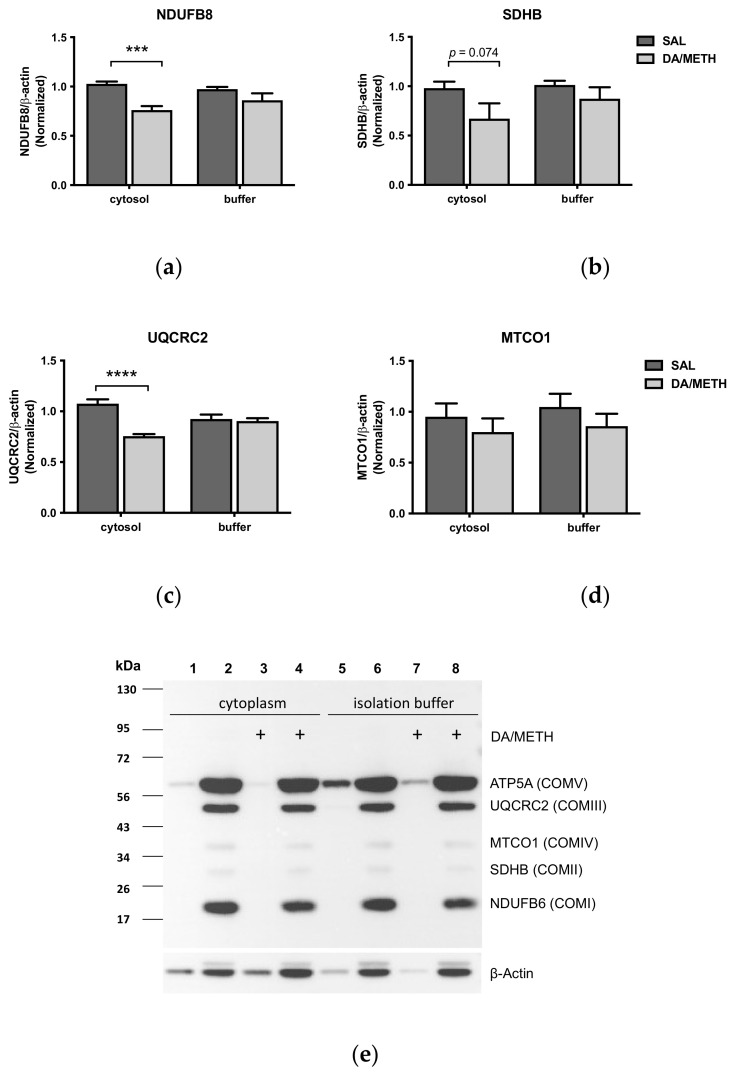
Effects of 10 mM DA and 10 µM METH on NDUFB6 (complex I), SDHB (complex II), UQCRC2 (complex III), MTCO1 (complex IV), and ATP5A (complex IV) in cytoplasm-suspended or buffer-suspended mitochondria. The DA and METH combination significantly decreased immunoreactivity of (**a**) NDUFB6, (**c**) UQCRC2, and (**e**) ATP5A in cytoplasm-suspended mitochondria. There was a trend toward statistical significance for a decrease in SDHB immunoreactivity (**b**) and no significant change in MTCO1 immunoreactivity (**d**) in these preparations. No significant changes were observed in enzyme immunoreactivities in buffer-suspended mitochondria. *** *p* < 0.001, **** *p* < 0.0001.

DA- or METH-induced ROS or RNS could have induced oxidative damage to multiple ETC subunits. To investigate whether other than already assessed ETC enzyme subunits are decreased by DA and/or METH, cytosol-suspended and isolation-buffer-suspended mitochondria were incubated with a mixture of 10 µM METH and 10 mM DA or saline for 30 min at 37 °C. The DA and METH combination caused a significant decrease in the immunoreactivity of the complex I subunit NDUFB8 (−28%, *p* < 0.001) complex III subunit UQCRC2 (−36%, *p* < 0.0001), and complex V subunit ATP5A (−26%, *p* < 0.01) (Student’s *t*-test) and produced a trend for statistical significance for a decrease in complex II subunit SDHB (−23%, *p* = 0.074) (Figure 5). In isolation-buffer-suspended mitochondria, the DA and METH mix did not significantly change the immunoreactivity of any of the subunits (Figure 5). These results suggest that multiple subunits ETC complexes are affected by DA and/or METH as early as 30 min after treatment and confirm that cytoplasmic components, such as degradation machinery, are necessary to observe deficits in the ETC complex subunits after exposure to DA or METH.

### 2.5. Effects of Dopamine and Methamphetamine Combination on Mitochondria from Striatal Synaptosomes

**Figure 6:**
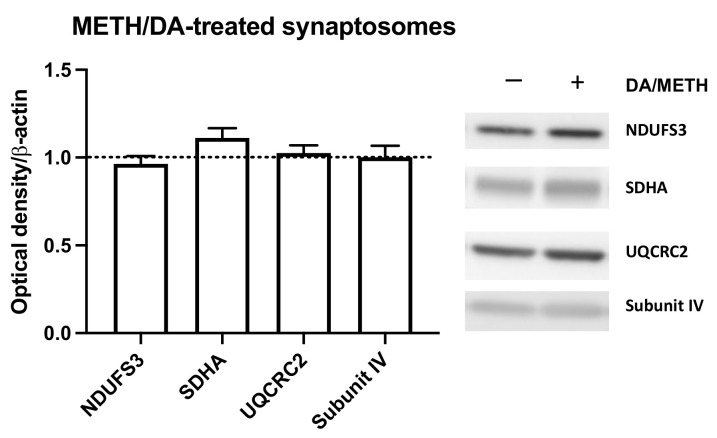
Effects of 10 mM DA and 10 µM METH on synaptosomal mitochondria: subunit NDUFS3 (complex I), SDHA (complex II), UQCRC2 (complex III), and subunit IV (complex IV). No statistically significant changes were observed in DA- and METH-treated synaptosomal mitochondria suspended in the cytoplasm compared to phosphate-buffered saline (PBS)-treated mitochondria. DA and METH data were normalized to the PBS data, shown as a dotted line.

The striatal mitochondria assessed above compromised a mixture of synaptosomal, neuronal, and glial mitochondria, with synaptosomal mitochondria constituting a small percentage of the overall mitochondrial population. To determine whether the DA and METH combination alters the immunoreactivity of ETC complexes in striatal synaptosomes, cytoplasm- or buffer-suspended synaptosomal mitochondria were treated with saline or with 10 µM METH and 10 mM DA combination for 30 min at 37 °C. The DA/METH mix had no effect on any of the examined subunits, namely NDUFS3, SDHA, UQCRC2, or subunit IV of complexes I, II, III, and IV, respectively (Figure 6).

### 2.6. Effects of Dopamine and Methamphetamine on Levels of the Protein Parkin

The protein parkin is a cytoplasmic protein involved in maintaining mitochondrial homeostasis. We previously showed that systemically administered binge METH decreases the levels of parkin in striatal synaptosomes via oxidative damage [24]. To determine the effects of increasing DA concentrations on parkin levels in perikaryal mitochondria, cytoplasmic and mitochondrial fractions were assessed for parkin levels after incubation with saline or DA (0.2, 0.8, 1, 5, 10, 20, or 50 mM) for 30 min at 37 °C. The immunoreactivity of parkin in the cytoplasmic fraction was significantly decreased at 5, 10, 20, and 50 mM DA (5 mM: −26%, *p* < 0.01; 10 mM: −29%, *p* < 0.05; 20 mM: −48%, *p* < 0.001; 50 mM: −71%, *p* < 0.001, one-way ANOVA, followed by Dunnett’s multiple comparison *post hoc* test) (Figure 7a). The decreases in the cytoplasmic parkin immunoreactivity were accompanied by increases in the immunoreactivity of parkin in the mitochondrial fraction, which reached statistical significance at 20 mM and 50 mM DA (20 mM: 2.2-fold, *p* < 0.05; 50 mM: 2.9-fold, *p* < 0.001; one-way ANOVA, followed by Dunnett’s multiple comparison *post hoc* test) (Figure 7b).

Damaged parkin aggregates after exposure to DA-induced oxidative stress [33]. To confirm that high DA concentrations induce aggregation of parkin, cytoplasm-suspended striatal mitochondria were incubated for 30 min at 37 °C with DA or with saline. These samples were then subjected to SDS-PAGE under reducing or nonreducing conditions. As expected, the nonreducing SDS-PAGE revealed cytoplasmic parkin immunoreactivity at ~52, ~70, and ~110 kDa in control samples (0 mM DA) and decreases in parkin immunoreactivity at these molecular weights after incubation with 20 mM DA. In the mitochondrial fractions run under the nonreducing conditions, parkin immunoreactivity was detected at ~52 kDa (the weakest), ~110 kDa, and above 120 kDa in control samples (0 mM DA). After treatment with 20 mM DA, parkin immunoreactivity increased in intensity at these molecular weights. Quantification of the 52 kDa band detected a significant increase in monomeric parkin immunoreactivity after incubation with 20 mM DA (~2.5-fold, *p* < 0.05, Student’s *t*-test) (Figure 7c,d).

Combined parkin immunoreactivity (total parkin) started to decrease at 10 mM DA (−23%) but reached statistical significance only at 50 mM DA (−43%, *p* < 0.01) (Figure 7e).

To determine the effects of METH alone on the immunoreactivities of striatal parkin, cytoplasm-suspended mitochondria were incubated with 10 µM METH for 30 min at 37 °C. A trend toward statistical significance for a decrease in parkin immunoreactivity was observed (−33%, *p* = 0.066, Student’s *t*-test) (Figure 7f). Neither 10 mM DA nor 10 µM METH decreased parkin immunoreactivity in isolation-buffer-suspended mitochondria (Figure 7g,h).

### 2.7. Effects of Parkin Overexpression and Binge Methamphetamine on Levels of NDUFS3 Subunit

**Figure 8:**
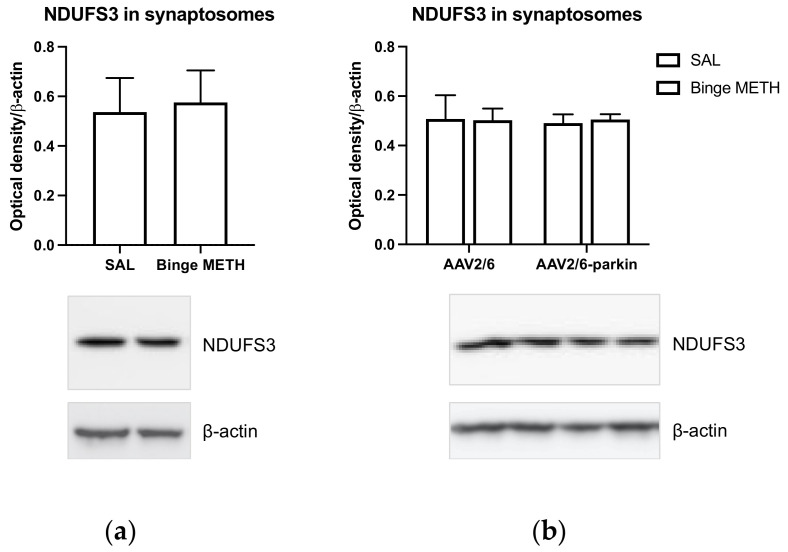
Effects of parkin overexpression and binge METH on the levels of NDUFS3 subunit in striatal synaptosomal mitochondria. (**a**) Compared to saline, binge METH (4 × 10 mg/kg, i.p., every 2 h) did not decrease NDUFS3 levels 1 h after the last injection of the drug. (**b**) A several-fold overexpression of parkin had no effect on NDUFS3 levels in saline-treated or METH-treated rats 1 h after the last injection of saline or METH.

Parkin dysfunction, as well as complex I dysfunction, has been implicated in the pathogenesis of PD [22,23]. Loss of parkin activity has been reported to cause deficits in respiratory complexes, particularly in complex I [21]. To determine whether systemically administered binge METH decreases complex I NDUFS3 levels in striatal synaptosomes in vivo and whether this effect can be rescued by parkin overexpression, rats overexpressing parkin in the nigrostriatal DA pathway were treated with binge METH (4 × 10 mg/kg, i.p., every 2 h) or saline (1 mL/kg) and sacrificed 1 h after the last injection of the drug or saline. Striatal synaptosomes were isolated and assessed for NDUFS3 immunoreactivity. Noncoding adeno-associated viral 2/6 vector (AAV2/6 vector, a control for AAV2/6-*parkin*) had no statistically significant effect on NDUFS3 immunoreactivity. Binge METH had no statistically significant effect on NDUFS3 immunoreactivity either in wild-type rats or in rats microinjected with noncoding AAV2/6. Finally, parkin overexpression (~4–7-fold) had no effect on NDUFS3 immunoreactivity either in saline- or METH-treated rats 1 h after the treatment. The data are summarized in Figure 8.

## 3. Discussion

In this study, we showed that high doses of DA alone rapidly (within 30 min) decrease the levels of complex I NDUFS3 subunit, whereas 10 µM METH alone rapidly decreases the levels of the complex I NDUFS3, complex II SDHA, and complex III UQCRC2 subunits in cytoplasm-suspended striatal mitochondria. We also showed that exposure of synaptosomal mitochondria to DA and METH does not lead to decreases in the levels of these mitochondrial subunits in vitro. Finally, we demonstrated that parkin levels and parkin overexpression do not influence the levels of the NDUFS3 subunit in vivo.

Mitochondrial function and integrity are crucial for cell survival [34]. Cytochrome c is a small hemeprotein primarily known as an electron carrier between mitochondrial complex III and complex IV and for its function in the intrinsic type II apoptosis [35]. Cytochrome c is released from mitochondria when proapoptotic stimuli induce the permeabilization of the outer mitochondrial membrane (OMM) and promote membrane depolarization [36]. Hence, mitochondrial membrane potential and cytochrome c release can serve as indicators of mitochondrial integrity and functionality [36,37]. Our finding that cytochrome c is released from cytoplasm- or isolation-buffer-suspended mitochondria after 30 min incubation with 10–50 mM DA or 10 µM METH proved that both molecules opened the mitochondrial permeability transition pores and depolarized the mitochondrial membrane in our mitochondrial suspensions. The lower increases in cytochrome c levels in the cytoplasm than in the buffer could have been due to degradation of cytochrome c by the cytoplasmic proteasome [38], which was not present in the buffer.

Our finding that METH alone decreased the levels of complex I NDUFS3 subunit, complex II SDHA subunit, and complex III UQCRC2 subunit, whereas DA alone decreased the levels only of complex I NDUFS3 subunit in cytoplasm-suspended striatal mitochondria, suggests that exposure to METH created a different microenvironment than exposure to DA. One possibility is that METH and DA may have had differential effects on the levels of mitochondrial subunits because they triggered the production of different ROS/RNS or the same ROS/RNS but at different levels or at different times. Available literature data indicate that exposure of mitochondria to micromolar concentrations of METH or millimolar concentrations of DA in vitro induces oxidative stress within 5 min [39,40] and that both molecules produce similar ROS/RNS within mitochondria. Thus, DA autoxidation (outside mitochondria and within mitochondria after sodium-ion-mediated transport) [41] likely generated hydrogen peroxide, DA quinone and semiquinone, and superoxide free radical, accompanied by the production of nitric oxide [40,42,43,44]. Some investigators have indicated that the source of nitric oxide in mitochondria is nitric oxide synthase located in this organelle in some cell types [45,46,47]; however, existing evidence suggests that this is not the case in neurons [48]. Consequently, NO levels in our mitochondrial suspensions were likely at baseline levels. The reaction of hydrogen peroxide with ferrous iron further produced hydroxyl free radical, while the reaction of nitric oxide with superoxide free radical may have produced peroxynitrite [49]. Both molecules easily cross phospholipid membranes and induce lipid peroxidation [39]. A byproduct of lipid peroxidation is 4-hydroxynonenal, which can decrease the activity of selective ETC subunits by covalent binding [50,51]. Oxidative damage to mitochondria by ROS most likely led to electron leakage and superoxide free radical production by complexes I and III [52]. METH is a cationic lipophilic molecule that can diffuse to mitochondria and be retained by this organelle [7]. Accumulation of positively charged molecules in mitochondria ultimately leads to dissipation of the electrochemical gradient, increased production of superoxide free radical by complexes I and III, cytochrome c release, and a decrease in ATP synthesis [7,13]. Mitochondrial manganese superoxide dismutase catalyzes the dismutation of superoxide radical to hydrogen peroxide [13]. These data indicate that incubation of mitochondria with METH likely produced superoxide and hydroxyl free radical, hydrogen peroxide, nitric oxide, and peroxynitrite. This conclusion is supported by the finding that exposure of non-DAergic cells to METH was followed by the production of hydrogen peroxide, superoxide and hydroxyl free radical, and protein nitrosylation [39,53]. What could explain our results is the potential differential binding of DA quinones and METH to the ETC subunits via sulfhydryl groups [39,54], damaging or obscuring epitopes for antibody binding. Along these lines, METH, but not DA quinones, could have bound to SDHA and UQCRC2 subunits. In support, Mashayekhi and colleagues found that METH could interact with respiratory complexes II and III, but not with complexes I and IV, in isolated liver mitochondria [39], whereas proteomic analysis determined that DA quinone bound to rat striatal complex I subunits 75 kDa and 30 kDa (the latter most likely NDUFS3) and complex III subunits (UQCRC1 and Rieske Fe-S protein), but not to other ETC subunits [55,56]. Of note, ROS can also bind to ETC subunits, causing oxidative damage, followed by the inaccessibility of epitopes to antibodies or transport from mitochondria and degradation in the cytoplasm [32,57,58].

We did not observe deficits in subunit IV or MTCO1 of complex IV either after METH or DA exposure. This is in contrast to a finding of decreased complex IV subunit II levels in T cells [53], decreased complex IV activity in rat striatum [59] and prefrontal cortex [60] following METH administration (50 µM for 24 h, 4 × 10, and 4 × 5 mg/kg, respectively), or decreased complex IV activity following exposure of mouse brain mitochondria to 1 mM DA [40]. However, similarly to our finding, the exposure of liver mitochondria to 5–20 µM METH or mouse brain mitochondria to 1–10 µM METH did not decrease complex IV activity [39,61], neither did the exposure of mice to 2 × 10 or 2 × 20 mg/kg METH 5 days after treatment [61]. A likely explanation for the discrepancy in the results is differences in the levels of nitric oxide at the times of measurement. Complex IV can be inhibited by nitric oxide; however, it is relatively more resistant to oxidative injury compared to other ETC complexes [62] while complexes I–II are insensitive to nitric oxide but susceptible to damage by ROS [63,64]. In vivo, glutamate is the main factor mediating induction of inducible nitric oxide synthase and production of nitric oxide in response to METH [7]. Glutamate was not present in the mitochondrial preparations in vitro. An alternative explanation for our observation is that complex IV activity decreased without significant changes in subunit IV or MTCO1 levels. Additional factors that could have induced differences in experimental findings are species, tissue type, dose, incubation time, and time of measurement after the treatment, among others.

The finding that the DA and METH combination significantly decreased the immunoreactivity of additional subunits, i.e., NDUFB6 of complex I, SDHB of complex II, and ATP5A of complex V, suggests that this combination oxidatively damages multiple ETC subunits, which agrees with available literature data. Literature data indicate that multiple subunits of all five ETC complexes are decreased after DA or METH administration in vivo and in vitro. For example, systemic administration of binge METH to rats (4 × 5 mg/kg, 2 h apart) decreased the levels of the complex I NDUFB10 subunit (and its mRNA) in the prefrontal cortex 12 h later [60], while 4 × 10 mg/kg binge METH decreased the levels of complex I NDUFA10, NDUFB5, NDUFS2, and NDUFS7, as well as complex V ATP5J2, ATP6V1D, and ATPIFL, subunits in mouse brain 7 days later [65]. Exposure of cultured T cells to 50 µM METH for 3 h led to decreased levels of the complex I NDUFB8 subunit (protein and mRNA), complex II core protein 2, and the complex IV subunit II [53]. Disintegration of whole mitochondria is a less likely scenario to explain our results because we found unchanged VDAC levels after incubation with 1–20 mM DA or 10 µM METH.

Mitochondrial proteins are degraded either by mitochondrial proteases or by proteasome or lysosome in the cytoplasm [32,57,58]. It has recently been shown that the parkin/PINK1 duo regulates the turnover of selective ETC protein subunits, including the 30 kDa complex I subunit (likely ortholog of rat NDUFS3) in *Drosophila* via proteasomal or lysosomal degradation and via regulation of ETC mRNA translation [19,20]. Consequently, the lack of decreased ETC subunits in buffer-suspended mitochondria could have been due to the absence of the cytoplasmic degradation machinery, which can function in homogenates (e.g., [24,66]).

Literature data on the effects of systemic METH on activity of complex I are discrepant. The Yamamoto group reported decreases in complex II [67], complex III, and complex IV [59] activities, but not in complex I activity, in rat striatum in vivo shortly (1–2 h) after administration of binge METH. In contrast, other groups reported significant decreases in complex I activity 12 h to days after binge METH or after chronic METH [61,68,69]. Studies that investigated protein levels of ETC subunits have provided similarly discrepant results. In mouse striatum, Klongpanichapak and colleagues found decreased levels of complex I (subunit not specified) 24 h after chronic treatment with METH [70], whereas Choi and colleagues found decreases in multiple complex I and complex V subunits a week after binge METH [65]. Our group found decreased levels of NDUFS3 in rat striatum 5 days after chronic treatment with 10 mg/kg/day METH [10]. These in vivo data suggest that either longer withdrawal time or longer treatment with METH is necessary for development of a complex I deficit in vivo. We detected decreases in complex I subunits in the present study as soon as 30 min, likely because compensatory mechanisms present in vivo, e.g., increased mitochondrial biosynthesis and axonal transport, were not available in our preparations.

Compared to previously published results [42,71], striatal mitochondria in our preparations appeared to be somewhat resistant to DA effects, as judged by cytochrome c release. The reported IC_50_ for inhibition of mitochondrial respiration is ~7 mM DA in intact mitochondria [40,72,73]. Moderate DA concentrations (<0.3 mM) act via a monoamine oxidase (MAO)-dependent mechanism, whereas at higher DA concentrations (>3 mM), the inhibition of mitochondrial respiration is increasingly MAO independent [42,72]. The MAO-independent component of mitochondrial inhibition by DA was reported to be 45, 68, and 100% at 5, 10, and 15 mM, respectively [72]. The relative “resistance” of our mitochondria to DA-induced oxidative stress could have been due to differences in suspension media composition (e.g., pH, ionic strength, redox state of DA), which can affect the rate of DA uptake by mitochondria [41,73] and differences in mitochondria themselves as they can respond differently to stressors depending on the source (e.g., species, tissue, brain area) [74]. At 50 mM DA, mitochondria were most likely disintegrating, as suggested by decreases in immunoreactivities of all mitochondrial proteins, including immunoreactivity of the VDAC, the abundant housekeeping mitochondrial protein [75].

The effects of METH or DA on synaptosomal mitochondria have not been extensively studied. Deficits in complex I subunits (NDUFS1, NDUFV1, and NDUFS5), complex III subunits (UQCRC1 and UQCRFS1), the complex IV subunit MTCO2, and complex V subunits (ATP5A and ATP1A3) were detected in rat striatal synaptosomes 14 days after chronic METH self-administration [11]. In contrast, increases were detected in complex I subunits (NDUFB3, NDUFB10, and NDUFS2) and complex V subunits (ATP5A1, ATP5F1, and ATFO) after acute METH treatment in synaptosomes from mouse brain [9]. Our group found no change in NDUFS3 and an increase in SDHA subunit in striatal synaptosomes in late-adolescent rats 1 h after binge METH [12] and decreases in both subunits 5 days after chronic METH administration [10]. In this study, we found no significant decreases in NDUFS3, SDHA, UQCRC2, or subunit IV in rat synaptosomes treated with DA and METH in vitro. Altogether, the data suggest that striatal synaptosomal mitochondria are potentially more “resistant” to oxidative damage than striatal mitochondria in neurons and glia in rats in that a chronic treatment with the drug appears to be needed to observe decreases in ETC subunit levels in synaptosomes, while several doses METH administered on the same day are enough to decrease ETC enzyme levels or activities in preparations from the whole striatum. Striatal synaptosomes are not a homogenous population. They consist of glutamatergic and monoaminergic synaptosomes, with glutamatergic synaptosomes being in a large majority [76]. Therefore, mitochondria in DAergic synaptosomes/terminals could have lost NDUFS3, SDHA, and UQCRC2 subunits as they experienced severe oxidative stress, but not glutamatergic synaptosomes, which are not affected by high doses of METH [77]. Against this hypothesis, Choi and colleagues found no difference between DAergic and non-DAergic mitochondria in sensitivity to stresses [78].

Parkin is a neuroprotective protein involved in maintenance of healthy mitochondria [18] and localizes mainly in the cytoplasm [79]. In vivo, high doses of METH oxidatively damage parkin and decrease its levels in striatal synaptosomes [79]. Consistent with that finding, in the present study, we observed a deficit in parkin in the cytoplasmic fraction. Parkin is susceptible to DA-mediated oxidative stress and prone to aggregation [33,80]. Therefore, the observed deficit in immunoreactivity of cytoplasmic parkin could have been due to DA-mediated oxidative damage to the protein (via covalent binding of DA quinone [33] or hydroxynonenal, a byproduct of lipid peroxidation [24]), aggregation, and degradation by the proteasome [24,33,80,81] or lysosome [82]. This explanation is consistent with the current observation that cytosolic parkin was not decreased by DA in isolation-buffer-suspended mitochondria. In the mitochondrial fraction, immunoreactivity of parkin increased and was distributed between monomeric parkin and parkin-positive bands of higher molecular weights. Endogenous parkin is located inside mitochondria, albeit at low levels, in different cell types, including neurons [19,20,83]. As such, parkin could have aggregated in DA-stressed mitochondria following binding of DA quinone or 4-hydroxynonenal [24]. The reasons for the increase in parkin levels in the mitochondrial fraction after DA and for the decrease after METH are not clear at present. The former may reflect translocation and binding of parkin to mitochondria in response to DA-induced oxidative stress caused by these molecules [84].

Inactivation of the *parkin* gene results in ETC function deficits, particularly in complex I [21,85], e.g., decreased abundance of several mitochondrial proteins, including the 24 and 30 kDa subunits of complex I in the mouse midbrain [49,85,86]. In the *Drosophila* brain, the turnover of mitochondria-selective ETC subunits at the OMM, including the 30 kDa subunit of complex I (likely an ortholog of rodent 30-kDa NDUFS3 subunit), occurs via proteasomal or lysosomal degradation and via regulation of ETC mRNA translation and is mediated by proteins parkin and PINK1 [19,20]. We tested a hypothesis that parkin regulates NDUFS3 levels in rat brain. Parkin overexpression in the nigrostriatal pathway did not change the levels of NDUFS3 in striatal synaptosomes from saline- or binge METH-treated rats, suggesting that parkin does not participate in turnover of this subunit in rat striatum.

**Figure 9:**
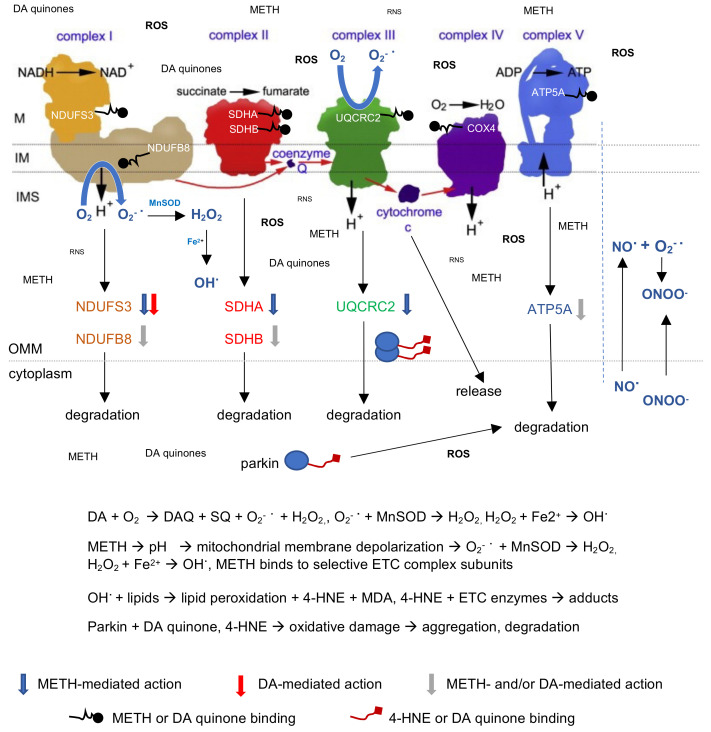
A model of DA and METH effects in cytoplasm-suspended mitochondria, based on our study data and the literature data. Dopamine (DA) autoxidation produces reactive oxygen species (ROS: O_2_^−^, H_2_O_2_, OH) and DA quinones, which bind to selective electron transport chain (ETC) enzyme subunits and trigger depolarization of mitochondria. Methamphetamine (METH) alkalization of the mitochondrial matrix depolarizes the mitochondrial membrane, leading to production of ROS. METH also binds to selective ETC enzyme subunits. Oxidatively damaged subunits are transported to the cytoplasm for degradation. Mitochondria contain nitric oxide (NO^·^), which produces another reactive nitrogen species (RNS), peroxynitrite (ONOO^−^), upon reaction with superoxide free radical (O_2_^−^^·^). RNS levels are low compared to ROS levels in vitro because of the absence of glutamate-induced generation of nitric and peroxynitrite (shown right from the dotted line). DA or METH administration triggers a release of cytochrome c. Hydroxyl free radical (OH^·^) induces lipid peroxidation with the production of 4-hydroxynonenal (4-HNA) and malondialdehyde (MDA). DA quinone and/or 4-HNE covalently binds to the protein parkin in the cytoplasm, leading to its aggregation and degradation. In mitochondria, aggregated parkin accumulates. Abbreviations: COX4, subunit IV of complex IV; IM, inner membrane; IMS, intramembrane space; M, matrix; mtNOS, mitochondrial nitric oxide synthase; OMM, outer mitochondrial membrane.

The neurotoxic effects of METH are of strong concern, and exploration of the mechanisms underlying this neurotoxicity has become a focus of research in recent years. The present results add to the existing knowledge in the area of METH neurotoxicity. Figure 9 presents a model of METH and DA effects on striatal mitochondria. From a public health point of view, the most important finding of the present study is that METH itself can affect the function of striatal mitochondria. This suggests that METH itself can be a drug target against its neurotoxicity in the striatum.

## 4. Materials and Methods

### 4.1. Animals

Male Sprague–Dawley rats (PND 55–65 upon arrival) were pair-housed under a 12 h light/dark cycle in a temperature-controlled (20–22 °C) and humidity-controlled room. Food and water were available ad libitum. After 1 week of acclimation, the rats were sacrificed or treated with binge METH. All animal procedures were conducted between 7:00 a.m. AND 7:00 p.m.

### 4.2. Preparation of Mitochondrial Suspensions

Mitochondria were isolated from fresh rat striata using the Mitochondria Isolation Kit for Tissue (Thermo Fisher Scientific, Waltham, MA, USA) according to the manufacturer’s instructions. Briefly, striata were homogenized in glass homogenizers, using a Dounce glass tissue grinder with a Teflon pestle (Wheaton Industries, Inc., Millville, NJ, USA) in an ice-cold isolation buffer supplemented with 4 mg/mL bovine serum albumin (BSA) and EDTA-free protease inhibitor cocktail (HALT). The homogenates were centrifuged at 800× *g* for 10 min at 4 °C, and mitochondria-containing supernatants were collected. Half of the obtained mitochondrial suspensions (cytoplasm-suspended mitochondria) were treated with saline, DA, or METH. The remaining half was centrifuged at 12,000× *g* at 4 °C for 15 min to separate mitochondria from the cytoplasm. The resulting mitochondrial pellet was extensively washed, resuspended in the isolation buffer (buffer-suspended mitochondria), and treated with saline, DA, or METH. All samples were kept on ice at all times between treatments.

### 4.3. Preparation of Synaptosomal Suspensions

Synaptosomal fractions were prepared via a differential centrifugation protocol as described previously [24]. Briefly, dissected out striata were manually homogenized in ice-cold 0.32 M sucrose supplemented with EDTA-free protease inhibitor cocktail (HALT) using a Dounce glass tissue grinder with a Teflon pestle (Wheaton Industries, Inc., Millville, NJ, USA), centrifuged once at 800× *g* for 24 min and then at 22,000 × *g* for 17 min (at 4 °C) to obtain the crude synaptosomal pellet. The pellet was resuspended in ice-cold distilled deionized water (to break synaptosomes). Subsequently, an equal volume of double-strength PBS was added to the suspension. Synaptosomal suspensions were treated with saline or the DA and METH combination.

### 4.4. Dopamine and Methamphetamine Treatment

In vivo, mitochondria within DAergic terminals are exposed to both METH and increasing concentrations of METH-released DA [87]. Synaptic METH concentration in the striatum shortly after its systemic binge administration (4 × 10 mg/kg) was reported to be 7–10 µM [88]. METH quickly enters the DAergic terminal via DAT and releases DA from the storage vesicles. DA released from the storage vesicles is subsequently released to the synaptic cleft within a short period of time (~30–60 min) [89,90,91] via a reversed action of DAT [15,89]. Synaptic concentrations of released DA can range between 30 and 100 mM before it dissipates [41,92]. Released DA autoxidizes with the production of several ROS. To approximate these conditions, cytoplasm-suspended striatal mitochondria were incubated with an increasing millimolar concentrations of DA (0.2, 0.8, 1, 5, 10, 20, and 50 mM) or 10 µM METH for 30 min at 37 °C. Control samples were treated with saline. Isolation-buffer-suspended mitochondria were generated to eliminate the potential influence of cytoplasmic components. They were incubated with 10 mM DA or 10 µM METH for 30 min at 37 °C. The concentration of 10 mM DA was chosen, as this concentration induced changes in cytoplasm-suspended mitochondria without affecting mitochondrial integrity and because this particular DA concentration triggers a significant decrease in cytoplasmic parkin immunoreactivity to similar levels observed 1 h after binge METH administration (4 × 10 mg/kg) in vivo ([24] and the Results section (Section 2)). Incubation of samples at 37 °C instead of 39 °C eliminated the influence of METH-induced hyperthermia, which is a confounding factor in vivo, as it can generate ROS and affect mitochondria by itself. After incubation, the mitochondrial suspensions (in striatal cytoplasm or isolation buffer) were centrifuged at 12,000× *g* at 4 °C for 15 min. Supernatants were transferred to fresh Eppendorf tubes and stored at –80 °C. The mitochondrial pellets were washed extensively, gently sonicated (at 30% power for 3–5 s) in 40 µL of 1% SDS in the mitochondria isolation buffer, and stored at −80 °C. Cytoplasm- or PBS-suspended synaptosomal mitochondria were treated with a cocktail of 10 mM DA and 10 µM METH for 30 min at 37 °C. After incubation, the synaptosomal suspensions were stored at −80 °C.

### 4.5. Electrophoresis and Western Blotting

The Pierce BCA Protein Assay Kit (Thermo Fisher Scientific, Waltham, MA, USA) was used to determine protein content. Mitochondrial and cytoplasm/buffer fractions were adjusted to a concentration of 10 µg protein/16 uL using NuPage^®^ 1× LDS sample buffer (Life Technologies, Carlsbad, CA, USA), containing 3.5 µL of β-mercaptoethanol as a reducing agent, and denatured for 10 min at 70 °C. In nonreducing SDS-PAGE, the reducing agent was omitted. Samples (10 µg protein/lane) were loaded onto NUPAGE 4–12% Bis-Tris gels and run for 1 h at 150 V. In some experiments, 20 µg protein/lane was loaded. Proteins were transferred onto methanol-activated polyvinylidene difluoride (PVDF) membranes for 1.5 h at 0.2 A. The membranes were blocked for 1 h at room temperature with 5% nonfat dried milk dissolved in TBST (10 mM Tris, 150 mM NaCl, and 0.5% Tween-20) and cut into pieces for incubation with primary antibodies against proteins differing in molecular weights. Next, the membranes were incubated overnight (at 4 °C) with mouse antibody against the mitochondrial complex I NDUFS3 subunit (1:1000, ab110246, Abcam, Cambridge, MA, USA), the mouse antibody against mitochondrial complex II SDHA subunit (1:1000, ab14715, Abcam), the rabbit antibody against complex III UQCRC2 subunit (1:1000, ab14745, Abcam), rabbit antibody against subunit IV of complex IV (1:1000, C#4844, Cell Signaling Technology, Danvers, MA, USA), rabbit antibody against VDACs (1:1000, C#4661, Cell Signaling Technology), mouse antibody against cytochrome c (1:1000, ab110325, Abcam), mouse antibody against parkin (1:1000, Prk8, Cell Signaling Technology), or mouse antibody cocktail against complexes I–V subunits NDUFB8, SDHB, UQCRC2, MTCO1, and ATP5A, respectively (1 ug/mL, C#45-8099, Thermo Fisher Scientific). Incubation with primary antibodies was followed by incubation with appropriate HRP-conjugated secondary antibodies (1:8000, 1 h at room temperature). Blots were developed using enhanced chemiluminescence (ECL) detection reagents and visualized using the LAS4000 bioimager (GE Healthcare, Piscataway, NJ, USA). The data were expressed as relative optical density units on each gel normalized to controls. This approach normalized differences in development of the blot and across blots. Total protein content of each lane, assessed by Ponceau S (P3504, Sigma-Aldrich) or actin (1:1000, ab8229, Abcam) staining, was used as a loading control.

### 4.6. Overexpression of Parkin In Vivo

Rats were anesthetized with 4% isoflurane and maintained under anesthesia at 2% isoflurane. Noncoding or parkin-coding adeno-associated vector 2/6 (AAV2/6 or AAV2/6-parkin) was microinjected into the substantia nigra *pars compacta* at the concentration of 2 × 10^7^ TUs/side using stereotaxic surgery as previously described [25]. Maximal parkin overexpression was achieved 3 weeks after AAV2/6-parkin microinjection, at which point rats were treated with binge METH or saline.

### 4.7. Binge Methamphetamine Treatment In Vivo

(+)-METH hydrochloride (METH-HCl) (Sigma-Aldrich, St. Louis, MO, USA) was dissolved in isotonic saline (0.9% NaCl) to a concentration of 10 mg/mL. METH-HCl (10 mg/kg) or saline (1 mL/kg) was administered to rats every 2 h in four successive i.p. injections. Rats were sacrificed by decapitation 1 h after the last injection of METH or saline. The brains were subsequently removed, and striata were collected. The striata were immediately processed to generate mitochondrial or synaptosomal suspensions.

### 4.8. Statistical Analyses

The differences between cytoplasm-suspended mitochondria exposed to a range of DA concentrations were determined by one-way ANOVA, followed by Dunnett’s multiple comparisons *post hoc* test. The differences between buffer-suspended mitochondria exposed to saline, 10 mM DA, or 10 µM METH were determined by the Student’s unpaired two-tailed *t*-test. Synaptosomal data were analyzed by Student’s unpaired two-tailed *t*-test or two-way ANOVA, followed by the Holm–Sidak *post hoc* test. The criterion for statistical significance for all comparisons was set at *p* < 0.05. A *p*-value between 0.05 and 0.10 was considered a statistical trend toward significance.

## 5. Conclusions

1. METH itself is a factor promoting dysfunction of striatal mitochondria.
2. DA and METH decrease activities of the ETC complexes via oxidative damage to their subunits.
3. Parkin does not regulate NDUFS3 turnover in rat striatum.
4. Synaptosomal mitochondria may be somewhat “resistant” to DA/METH-induced disruption in mitochondrial ETC complexes than perikaryal mitochondria.

## Figures and Tables

**Figure A2:**
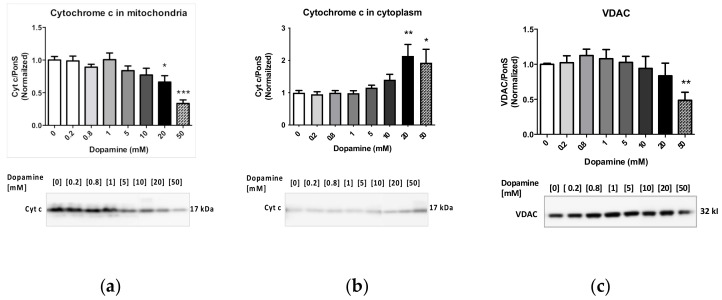
Effects of increasing doses of DA on the levels of cytochrome c (cyt c) in (**a**) mitochondrial fraction and (**b**) cytoplasmic fraction. (**c**) Effects of increasing doses of DA on the levels of the VDAC in mitochondrial fraction. * *p* < 0.05, ** *p* < 0.01, *** *p* < 0.001, one-way ANOVA with Dunnett’s *post hoc* test.

## Data Availability

Not applicable.

## Appendix A

### Appendix A.1. Integrity of Isolated Mitochondria

#### Appendix A.1.1. Incubation at 37 °C

To determine whether 30 min incubation by itself affected the integrity of mitochondria in our preparations, the levels of cytochrome c were assessed in mitochondrial and cytoplasmic fractions before and after 30 min incubation at 37 °C. As shown in Figure A1a, the immunoreactivity of cytochrome c in the cytoplasmic, as well as in the mitochondrial, fraction was the same before and after incubation, i.e., cytochrome c was not released from the mitochondria, indicating that cytoplasm-suspended mitochondria were intact over 30 min at 37 °C. Incubation of isolation-buffer-suspended mitochondria for 30 min at 37 °C without METH or DA treatment also did not result in cytochrome c leakage (Figure A1b), indicating that isolation-buffer-suspended mitochondria also remained intact during incubation. A weak band visible in some blots in nonmitochondrial fractions may represent slight mechanical damage to mitochondria during isolation. Weak bands observed at a molecular weight higher than 14 kDa in the mitochondrial fraction likely represent post-translational modifications of cytochrome c. A ~30 kDa band likely represents cytochrome c bound to subunit IV isoform 2 (COX4I2), subunit of complex IV [94,95], which has a molecular weight of ~20 kDa. VDAC is a multifunctional housekeeping mitochondrial protein, often used as a mitochondrial loading control due to its abundance. The immunoreactivity of the VDAC was also not changed by incubation, thus confirming that incubation itself does not compromise mitochondria (Figure A1a).

#### Appendix A.1.2. Dopamine Treatment

It has been shown that 0.2 mM DA has the ability to depolarize mitochondria with a consequent release of cytochrome c [29,30]. To determine DA concentrations at which cytoplasm-suspended mitochondria release cytochrome c, mitochondrial preparations were incubated with saline or DA at a concentration of 0.2, 0.8, 1, 5, 10, 20, or 50 mM for 30 min at 37 °C. The immunoreactivity of cytochrome c in mitochondrial preparations significantly decreased after 30 min incubation with 20 or 50 mM DA (−34%, *p* < 0.05 and −67%, *p* < 0.001, respectively, one-way ANOVA, followed by Dunnett’s multiple comparison *post hoc* test). Concomitantly, the immunoreactivity of cytochrome c in the cytoplasmic fraction increased (2.1-fold, *p* < 0.01 and 1.9-fold, *p* < 0.05, respectively, in relation to a weak cytochrome c-positive band in the cytoplasmic fraction; one-way ANOVA, followed by Dunnett’s multiple comparison *post hoc* test). The data are presented in Figure A2a,b.

To determine whether high concentrations of DA affect the levels of the VDAC, cytoplasm-suspended mitochondria were incubated without or with DA at a concentration of 0.2, 0.8, 1, 5, 10, 20, or 50 mM for 30 min at 37 °C. Incubation of mitochondria with 50 mM DA significantly decreased the immunoreactivity of the VDAC in the mitochondrial fraction (−51%, *p* < 0.001, one-way ANOVA, followed by Dunnett’s multiple comparison *post hoc* test) (Figure A2c), suggesting that at 50 mM DA, mitochondria disintegrate.

### Appendix A.2. NDUFS3, SDHA, and UQCRC2 Deficits Are Not Caused by a Leakage or Aggregation

#### Appendix A.2.1. Supplementary Data

The decreased immunoreactivity of the NDUSF3 observed after incubation of cytoplasm-suspended mitochondria with DA was not due to its leakage from mitochondria or its aggregation (and the consequent potential inability of the NDUSF3 antibody to reach its epitope) because NDUSF3 immunoreactivity was not detected in the cytoplasmic fraction or above 30 kDa when using nonreducing SDS-PAGE (Figure A3).

#### Appendix A.2.2. Supplementary Data

The decreased immunoreactivity of NDUSF3, SDHA, and UQCRC2 observed after incubation of cytoplasm-suspended mitochondria with METH was not due to their leakage from the mitochondria because NDUSF3, SDHA, or UQCRC2 immunoreactivity was not detected in the cytoplasmic fraction (reducing SDS-PAGE) (Figure A4).
